# A Gene Regulatory Network Controlled by BpERF2 and BpMYB102 in Birch under Drought Conditions

**DOI:** 10.3390/ijms20123071

**Published:** 2019-06-23

**Authors:** Xuejing Wen, Jingxin Wang, Daoyuan Zhang, Yucheng Wang

**Affiliations:** 1 CAS Key Laboratory of Biogeography and Bioresource in Arid Land, Xinjiang Institute of Ecology and Geography, Urumqi 830011, China; wxj-329@163.com (X.W.); Zhangdy@ms.xjb.ac.cn (D.Z.); 2 Turpan Eremophytes Botanical Garden, Chinese Academy of Sciences, Turpan 838008, China; 3 University of Chinese Academy of Sciences, Beijing 100049, China; 4 State Key Laboratory of Tree Genetics and Breeding, Northeast Forestry University, Harbin 150040, China; wjx206091@163.com

**Keywords:** transcription factor, transient transformation, drought stress, expression regulatory network, *Betula platyphylla*, RNA-Seq

## Abstract

Gene expression profiles are powerful tools for investigating mechanisms of plant stress tolerance. *Betula platyphylla* (birch) is a widely distributed tree, but its drought-tolerance mechanism has been little studied. Using RNA-Seq, we identified 2917 birch genes involved in its response to drought stress. These drought-responsive genes include the late embryogenesis abundant (LEA) family, heat shock protein (HSP) family, water shortage-related and ROS-scavenging proteins, and many transcription factors (TFs). Among the drought-induced TFs, the ethylene responsive factor (ERF) and myeloblastosis oncogene (MYB) families were the most abundant. BpERF2 and BpMYB102, which were strongly induced by drought and had high transcription levels, were selected to study their regulatory networks. BpERF2 and BpMYB102 both played roles in enhancing drought tolerance in birch. Chromatin immunoprecipitation combined with qRT-PCR indicated that BpERF2 regulated genes such as those in the *LEA* and *HSP* families, while BpMYB102 regulated genes such as *Pathogenesis-related Protein 1* (*PRP1*) and *4-Coumarate:Coenzyme A Ligase 10* (*4CL10*). Multiple genes were regulated by both BpERF2 and BpMYB102. We further characterized the function of some of these genes, and the genes that encode Root Primordium Defective 1 (RPD1), PRP1, 4CL10, LEA1, SOD5, and HSPs were found to be involved in drought tolerance. Therefore, our results suggest that BpERF2 and BpMYB102 serve as transcription factors that regulate a series of drought-tolerance genes in *B. platyphylla* to improve drought tolerance.

## 1. Introduction

Plant growth is greatly influenced by adverse environmental conditions, such as drought, salt, or extreme temperature. Among these adverse factors, drought stress is commonly encountered, and it is aggravated by climate changes, population growth, and the increase in water use. Therefore, to alleviate or solve the problems caused by drought, it is necessary to clarify the drought-tolerance mechanisms of plants.

Drought stress conditions trigger changes in the complex biological processes of plants at the molecular, physiological, and biochemical level. Of these changes, gene expression profiles are the first to be altered. These differentially expressed genes can be generally grouped into two classes. One class contains ‘regulatory proteins’ that play roles in signal transduction and regulate the expression of genes involved in the stress response; among this group are genes that encode signal proteins and transcription factors [1,2]. The other class includes genes that serve to protect plant cells from abiotic stress. These genes are usually involved in the following functional categories: chaperones, reactive oxygen species scavenging, stabilization of membranes, osmoprotectant biosynthesis, iron homeostasis, amino acid metabolites, sucrose transporters, phloem loading, and photosynthesis [2,3,4].

Gene expression profiles are frequently used to investigate the mechanisms of drought stress tolerance. For instance, Liu et al. [5] identified differentially expressed genes (DEGs) between control plants and PEG-treated *Reaumuria soongorica*, and they found 379 up-regulated genes and 946 down-regulated genes under drought stress conditions. Analysis of these DEGs showed that *R. soongorica* may adapt to drought stress conditions by inducing effective signal transduction pathways and increasing the protection of functional proteins to re-establish cellular homeostasis. Gene expression was compared between drought-tolerant and drought-sensitive maize lines under moderate drought, severe drought, and sufficient water (controls). Transcription factors were further analyzed between these two lines, and the genotype-specific response of TFs in the tolerant line and the sustained genotypically differential expression of TFs were concluded to potentially play important roles in the enhanced tolerance to drought in maize [6]. Kumar et al. [7] collected a series of genome-wide transcriptome data from *japonica* and *indica* rice cultivars under cold stress conditions. Analysis of these data revealed biological processes and related regulatory pathways in response to drought stress. From their results, they proposed a model that included a pathway for cold stress-responsive signaling to explain the gene expression profiles in sensitive and tolerant rice under drought stress conditions. Analysis of DEGs led to the identification of several shared and distinct biological processes between tolerant and sensitive varieties as well as candidate stress-responsive genes [8]. In addition, SNPs are important in the identification of genes contributing to abiotic stress tolerance. For instance, Xu et al. [9] compared 16 maize inbred lines and identified candidate nsSNPs and associated genes involved in drought tolerance. Dalal et al. [10] studied the molecular mechanism of drought-induced root growth in wheat using RNA-Seq. They identified 2783 and 2638 DEGs in two wheat genotypes—Raj3765 and HD2329—that differ in root growth under drought stress. Their studies suggested that drought-induced root growth in wheat requires a complex interplay between cell wall synthesis, cellular tolerance, hormones, and ROS metabolism. Fox et al. [11] investigated the dynamics of the molecular and physiological responses in *Pinus halepensis* under drought stress conditions, and transcriptome analysis was performed at six physiological stages. Their results showed that drought stress induced processes such as the abscisic acid response; ROS-scavenging through AsA-independent thiol-mediated pathways; accumulation of heat shock proteins, thaumatin, and exordium; and chlorophyll degradation. To alleviate the damage caused by drought, the drought-tolerant wheat cultivar JM-262 produces ROS scavengers, osmoprotectants, biomass, and energy under drought stress [12]. According to RNA-Seq studies, the response or tolerance to abiotic stress involves many transcription factor families, such as bZIP [13,14], NAC [14,15], ERF, HSF, ARF [6], AP2-DREB, WRKY, C2H2 [15], and trihelix [16]. RNA-Seq has been widely performed to reveal the expression of genes in response to different abiotic stresses on a genome scale, and its results facilitate the understanding of mechanisms involved in abiotic stress tolerance.

Although gene expression profiles have been built, the regulatory networks of these abiotic stress response genes are mostly unknown; in addition, the mechanisms of stress tolerance resulting from these stress-responsive genes have not been identified. In the present study, we used a very effective strategy to build the gene expression profile of birch (*Betula platyphylla*) in response to drought stress. From this profile, we identified the transcription factors involved in the drought stress response and determined the function of some of their target genes in drought tolerance. This study provides useful information for the characterization of drought tolerance in birch, and the strategy and method used in this study can contribute to the construction of a genetic regulatory network and the identification of stress tolerance genes on a large scale.

## 2. Results

### 2.1. The Physiological Changes in Birch in Response to Drought Stress

**Figure 1:**
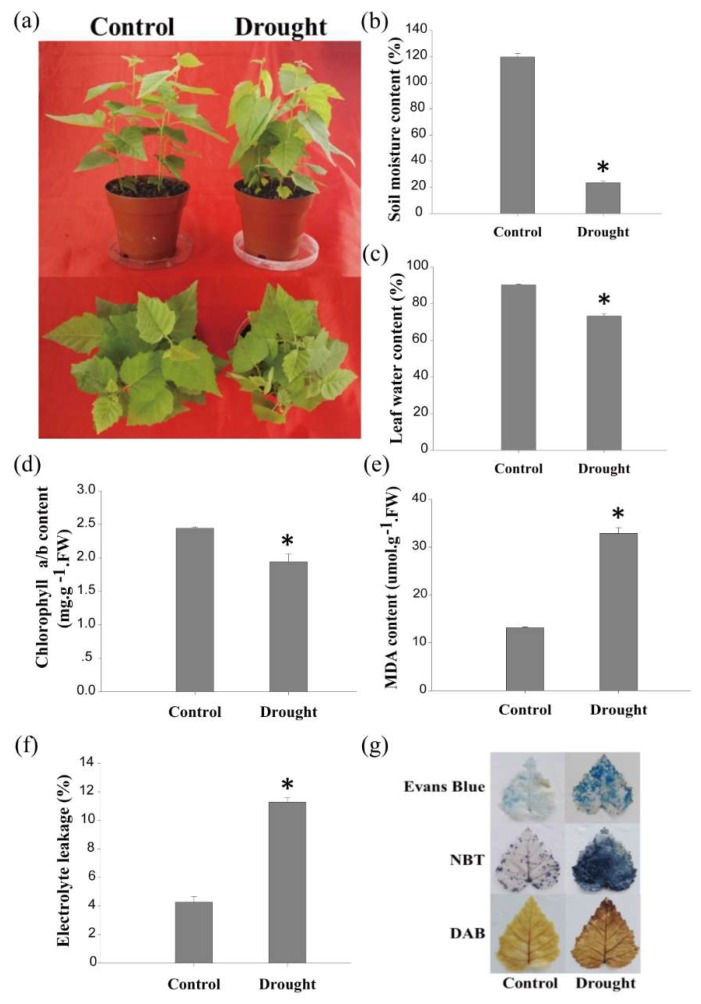
Drought treatment of birch plants. (**a**) The growth phenotype of birch under drought or normal (control) growth conditions. Watering of birch plants grown in soil was stopped for 120 h, and the well-watered birch plants served as the controls; (**b**) measurements of soil moisture; (**c**) leaf water content study; (**d**) measurements of total chlorophyll; (**e**) MDA content analysis; (**f**) electrolyte leakage assay; (**g**) NBT, DAB, and Evans blue staining for birch leaves under normal or drought stress conditions. FW: fresh weight. Asterisks indicate a significant difference between treatment and control plants (*p* < 0.05).

For the drought treatment, birch plants were not watered for 120 h. The drought-treated plants showed an injured phenotype when compared with the birch plants under normal conditions (control). The leaves of the drought-treated plants were drooped (Figure 1a), and the soil moisture content of the drought-treated plants decreased by 96% compared with that of the control plants (Figure 1b). Correspondingly, the water content in the birch leaves under drought conditions decreased by 17% when compared with that of the control plants (Figure 1c). The total chlorophyll content of the birch leaves under drought conditions decreased from 2.44 to 1.94 mg·g^−1^ (Figure 1d). Diaminobenzidine (DAB) and nitroblue tetrazolium (NBT) staining indicated that H_2_O_2_ and O^2−^ levels were dramatically increased under drought stress conditions (Figure 1g). Furthermore, the malondialdehyde (MDA) content in birch was significantly increased by drought stress (Figure 1e). Evans blue staining and the electrolytic leakage rate assay both showed that the cell membrane was damaged by drought stress (Figure 1f,g). These results indicate that naturally drying birch for 120 h triggered a significant drought response; thus, these drought-treated plants were suitable as material for further study.

### 2.2. Identification of DEGs in Response to Drought Stress in Birch

To survey the transcripts associated with the drought stress response on a genome scale in birch, six cDNA libraries were constructed from mRNAs isolated from birch after a 120-h drought and birch under normal conditions (three independent biological replications). In total, 39.40 Gb of clean nucleotide data were obtained from the six libraries. The Pearson’s correlation coefficient of three independent biological replicates under the same conditions was 0.868–0.981, indicating the repeatability of the study (Supplementary Figure S1a). The distribution of differentially regulated genes is visualized as a volcano plot (Supplementary Figure S1b). The results revealed a total of 2917 DEGs, including 1127 genes induced and 1790 genes inhibited by drought (Supplementary Table S4).

Among the 2917 DEGs, 2875 DEGs were functionally annotated using Gene Ontology (GO) analysis. In the biological process, the genes involved in the rhythmic process were highly enriched, but the genes related to the biological phase GO term were drastically reduced. In the cellular component, genes involved in the extracellular region, extracellular region part, extracellular matrix part, and nucleotide categories were all highly enriched. In the molecular function, the nucleic acid binding transcription factor, electron carrier, antioxidant, protein binding transcription factor, and guanyl-nucleotide exchange factor were all highly enriched (Supplementary Figure S1c).

**Figure 2:**
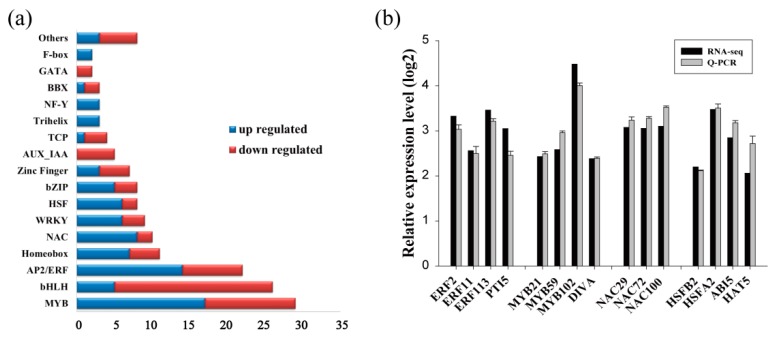

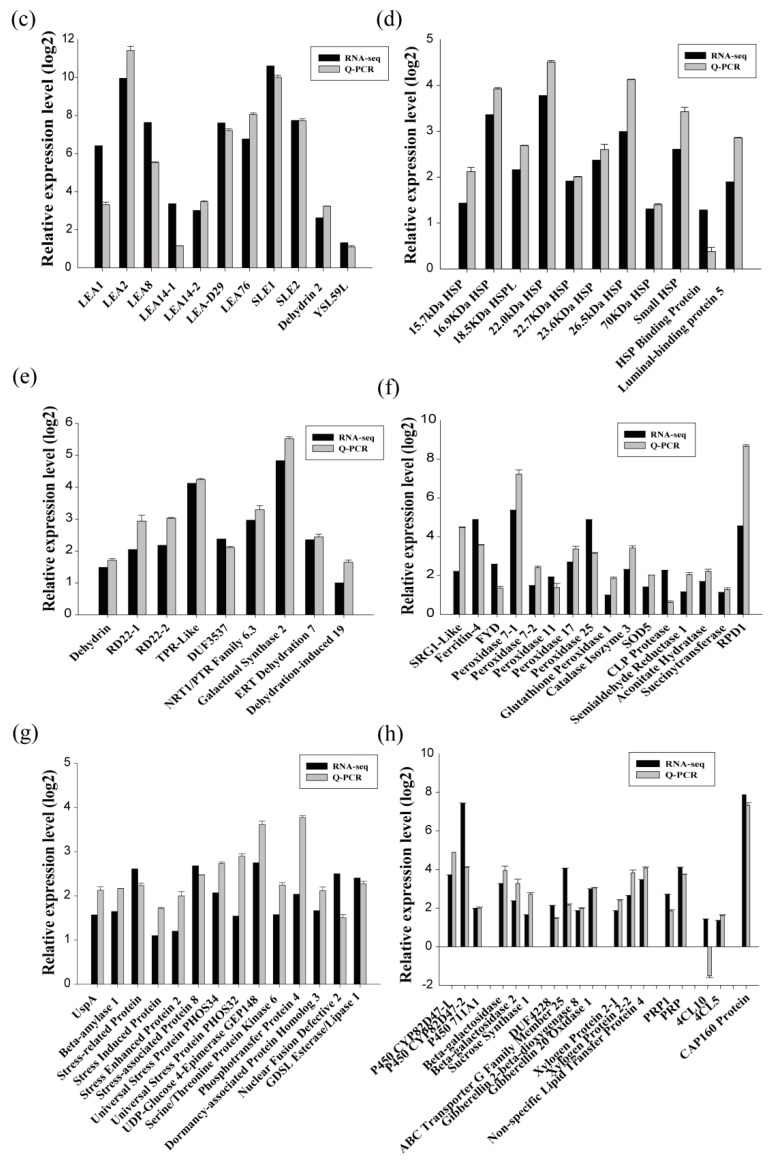
Quantitative RT-PCR confirmation of the differentially expressed genes identified using RNA-Seq. (**a**) The families of differentially expressed transcription factors in birch under drought stress conditions. (**b**–**h**) Comparison of the expression level of differentially expressed genes in the function involved in: transcription factors (**b**), late embryogenesis abundant proteins (**c**), heat shock proteins (**d**), ROS-scavenging-related proteins (**e**), water deprivation-related proteins (**f**), stress-related proteins (**g**), and others (**h**) between qRT-PCR and RNA-Seq.

Because transcription factors (TFs) play crucial roles in transcriptional regulation and the stress response, we further identified differentially expressed TFs: 160 TFs were found to be responsive to drought stress (Figure 2a), including 84 drought-induced TFs and 76 drought-down-regulated TFs. Among the TFs up-regulated by drought, the MYB and ERF families were the most abundant (with 17 and 14 DEGs, respectively), followed by the NAC (eight DEGs), Homeobox (seven DEGs), WRKY (six DEGs), and HSF (six DEGs) families. In the down-regulated TF families, the bHLH family was the most abundant (21 DEGs), followed by the MYB family (12 DEGs) and the ERF family (eight DEGs) (Figure 2a).

To evaluate the reliability of the data generated with RNA-Seq, qRT-PCR was performed, and 94 randomly selected DEGs, including 15 drought-up-regulated TFs, were analyzed. The results showed that the expression of most DEGs determined by qRT-PCR was consistent with those identified by RNA-Seq, confirming the reliability of the RNA-Seq results (Figure 2b–h). The results of qRT-PCR combined with those of RNA-Seq revealed many strongly induced stress-related genes, including the late embryogenesis abundant (LEA) protein family (Figure 2c), heat shock protein (HSP) family (Figure 2d), water deprivation-related genes (Figure 2e), ROS-scavenging-related genes (Figure 2f), and stress-related genes (Figure 2g). These results indicate that these genes may play important roles in the drought stress tolerance of birch plants.

### 2.3. Identification of the Transcription Factors Involved in the Tolerance to Drought Stress

**Figure 3:**
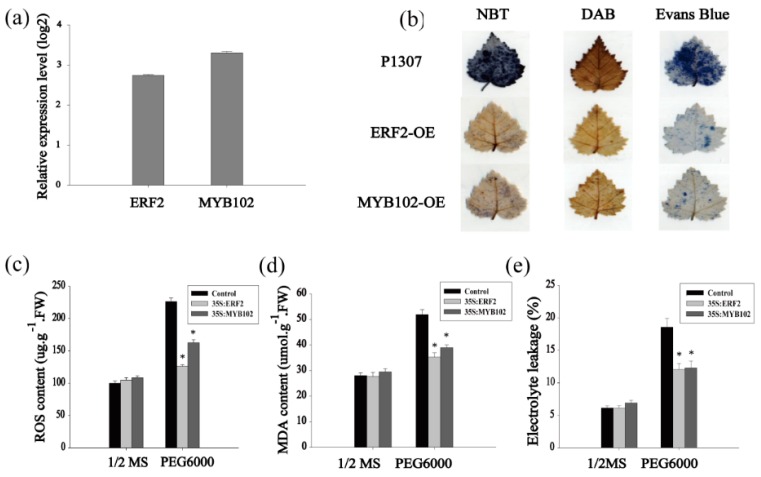
Analysis of drought stress tolerance of BpERF2 and BpMYB102 in birch. Three kinds of transgenic plants were compared: plants transiently transformed with 35S:BpERF2, 35S:BpMYB102, and empty p1307-Flag (as the control). (**a**) The study of the expression of BpERF2 and BpMYB102 in transiently transformed plants using qRT-PCR. (**b**) NBT, DAB, and Evans blue staining of leaves from the three kinds of plants. (**c**–**e**) Comparison of ROS content (**c**), MDA content (**d**), and electrolyte leakage (**e**) among the three kinds of plants under normal or drought stress conditions. 1/2 MS: the plants grown on 1/2 MS medium as the control; PEG6000: the plants grown on 1/2 MS medium supplying with 20% PEG6000, which is used as drought stress. Asterisks indicates a significant difference between treatment and control plants (*p* < 0.05).

The fact that many of the ERF and MYB family TFs were differentially regulated suggests that they may play roles in the drought stress response. We further studied the contribution to drought tolerance of some genes in the ERF and MYB families. Among them, *BpERF2* and *BpMYB102* were highly induced by drought and had high absolute expression values, so they were selected for further study. Two types of transformed birch plants were generated using the transient transformation method, i.e., control plants were transformed with empty p1307-Flag (control), and the plants transiently transformed with *35S:BpERF2* or *35S:BpMYB102* transiently overexpressed *BpERF2* or *BpMYB102*, respectively. The qRT-PCR results showed that the expression levels of both *BpERF2* and *BpMYB102* were markedly increased in the transgenic plants compared with the control plants (Figure 3a), indicating the successful generation of transiently transformed plants that overexpressed *BpERF2* and *BpMYB102*; thus, these plants were suitable for further study. To determine the physiological responses mediated by BpERF2 or BpMYB102, the ROS content, MDA content, and cell damage were measured. Under normal conditions, there were no obvious differences in these physiological responses between the plants that transiently overexpressed *BpERF2* or *BpMYB102* and the control plants. Under drought stress conditions, NBT and DAB staining showed that the plants that overexpressed *BpERF2* or *BpMYB102* both had reduced H_2_O_2_ and O^2−^ accumulation (Figure 3b). We further measured the ROS content of these plants using an enzyme linked immunosorbent assay (ELISA) method. Compared with the control plants, the plants overexpressing *BpERF2* or *BpMYB102* both showed significantly decreased ROS content under the drought stress condition (Figure 3c). Additionally, plants overexpressing *BpERF2* or *BpMYB102* also showed significantly decreased MDA content in comparison with the control plants under drought stress conditions (Figure 3d). At the same time, plants overexpressing *BpERF2* or *BpMYB102* displayed significantly decreased electrolyte leakage (Figure 3e). Consistently, cell death detected by Evans blue staining also showed that overexpression of BpERF2 or BpMYB102 substantially decreased cell membrane damage (Figure 3b). Taken together, these results indicate that the overexpression of BpERF2 or BpMYB102 could improve tolerance to drought stress, suggesting that they play positive roles in the improvement of stress tolerance.

**Figure 4:**
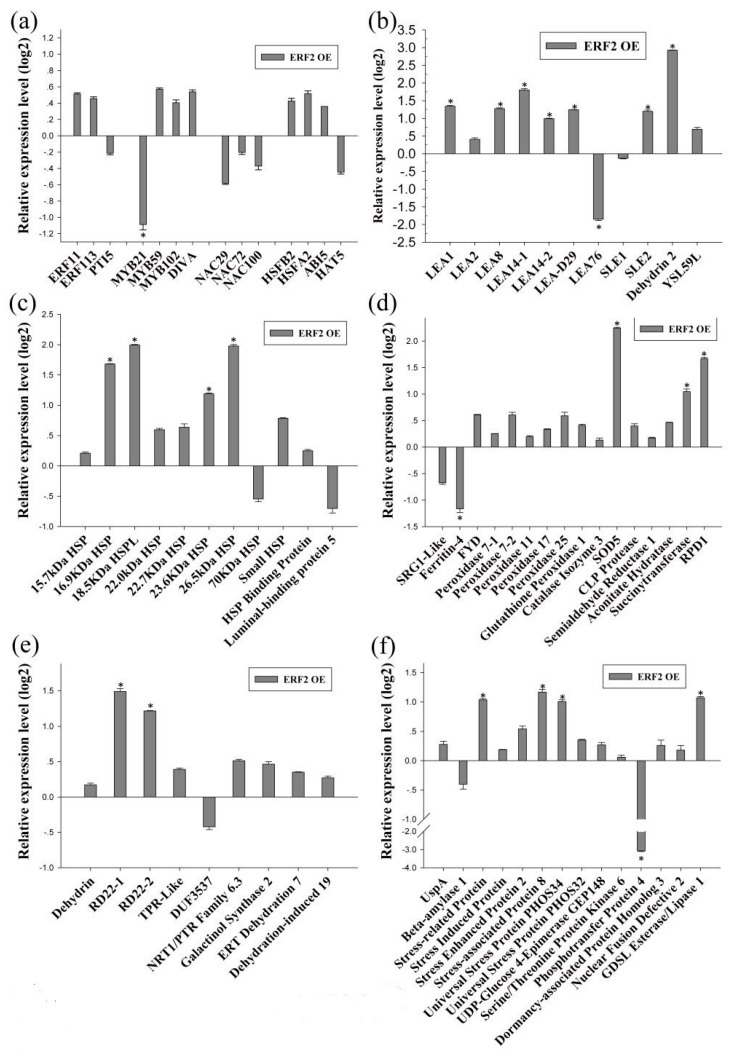

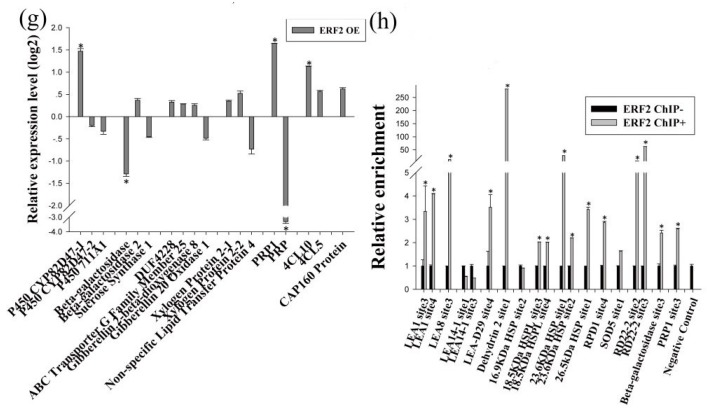
Determination of the genes regulated by BpERF2. (**a**–**g**) Investigation of the genes regulated by BpERF2 using qRT-PCR. Transcription factors (**a**), late embryogenesis abundant proteins (**b**), heat shock proteins (**c**), ROS-scavenging-related proteins (**d**), water deprivation-related proteins (**e**), stress-related proteins (**f**), and others (**g**). The transcripts of the genes in control plants (transiently transformed with empty p1307-Flag) were used to normalize their expression in the plants that transiently overexpressed BpERF2 according to qRT-PCR. (**h**) Investigation of whether BpERF2 directly regulates its target genes using ChIP. BpERF2 was fused with a Flagtag, transiently transformed into the plants, and used for ChIP. ChIP+: sonicated chromatin immunoprecipitated with anti-Flag antibody; ChIP−: sonicated chromatin immunoprecipitated without any antibody. Asterisks indicates a significant difference between treatment and control plants (*p* < 0.05 and |log2FC| ≥ 1).

As BpERF2 and BpMYB102 are transcription factors, we further determined their target genes using qRT-PCR. To this end, several genes that were potentially involved in the abiotic stress response were selected from the identified DEGs for further study. The results showed that overexpression of BpERF2 could significantly induce a series of genes involved in abiotic stress tolerance (Figure 4a–g), suggesting that these genes are directly or indirectly regulated by BpERF2. To further determine whether BpERF2 directly regulates these genes, ChIP-qPCR was performed. The results showed that BpERF2 was able to bind to the promoters of the birch genes homologous to *LEA1*, *LEA8*, *LEA-D29*, *Dehydrin 2*, *18.5 kDa heat shock protein like (HSPL)*, *23.6 kDa HSP*, *26.5 kDa HSP*, *Root Primordium Defective 1* (*RPD1*), *RD22-2*, *Pathogenesis-related Protein 1* (*PRP1*), and *Beta-galactosidase*, but it failed to bind to the promoters of other genes shown in Figure 4a–g (Figure 4h). Therefore, BpERF2 directly and indirectly regulated numerous genes, including LEA family members, HSP family members, ROS-scavenging-related genes, water deprivation-related genes, and stress-related genes.

**Figure 5:**
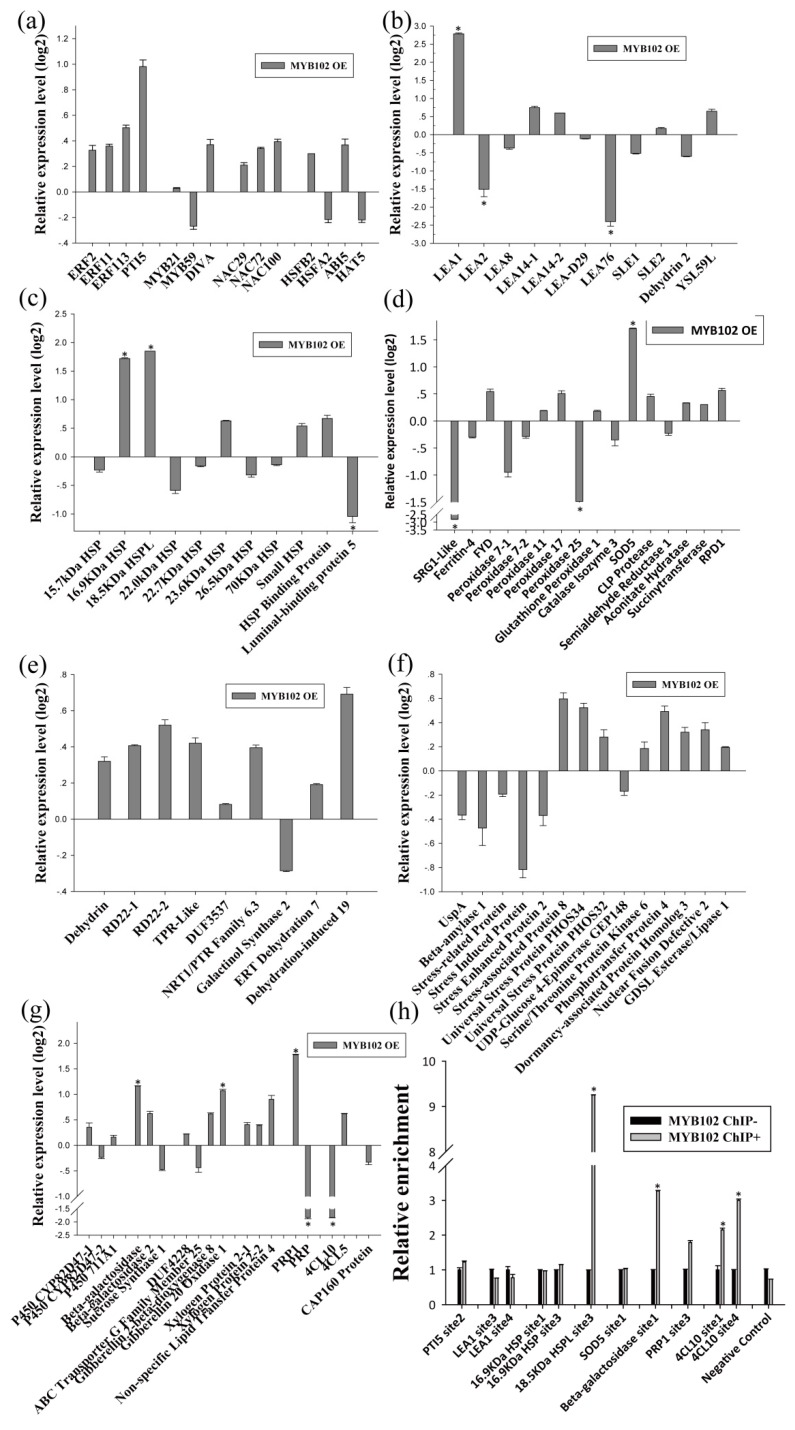
Determination of the genes regulated by BpMYB102. (**a**–**g**) Investigation of the genes regulated by BpMYB102 using qRT-PCR. Transcription factors (**a**), late embryogenesis abundant proteins (**b**), heat shock proteins (**c**), ROS-scavenging-related proteins (**d**), water deprivation-related proteins (**e**), stress-related proteins (**f**), and others (**g**). The transcripts of the genes in control plants (transiently transformed with empty p1307-Flag) were used to normalize their expression in the plants that transiently overexpressed BpMYB102 according to qRT-PCR. (**h**) Investigation using ChIP of whether BpMYB102 directly regulates its target genes. BpMYB102 was fused with a Flag tag, transiently transformed into the plants, and used for ChIP. ChIP+: sonicated chromatin immunoprecipitated with anti-Flag antibody; ChIP−: sonicated chromatin immunoprecipitated without any antibody. Asterisks indicates a significant difference between treatment and control plants (*p* < 0.05 and |log2FC| ≥1).

On the other hand, qRT-PCR showed that the birch genes homologous to *PTI5*, *LEA1*, *16.9 kDa HSP*, *18.5 kDa HSPL*, *SOD5*, *Beta-galactosidase*, *PRP1*, and *Gibberellin 20 Oxidase 1* were induced by BpMYB102, suggesting that they may be target genes of BpMYB102. In addition, qRT-PCR showed that *4-Coumarate-CoA Ligase 10* (*4CL10*) was significantly down-regulated in the plants overexpressing *BpMYB102*, indicating that BpMYB102 may negatively regulate the expression of *4CL10* (Figure 5g). Furthermore, the ChIP-qPCR results showed that BpMYB102 was able to bind to the promoters of *18.5 kDa HSPL*, *Beta-galactosidase*, and *4CL10*, suggesting that BpMYB102 can directly regulate these genes, but it did not bind to the promoters of the other asterisk genes shown in Figure 5a–g (Figure 5h). Notably, there was no detected regulatory relationship between BpERF2 and BpMYB102 (Figure 4a and Figure 5a), suggesting that they are likely involved in different regulatory networks.

### 2.4. Identification of the Drought Tolerance of the Genes Regulated by MYB and ERF

**Figure 6:**
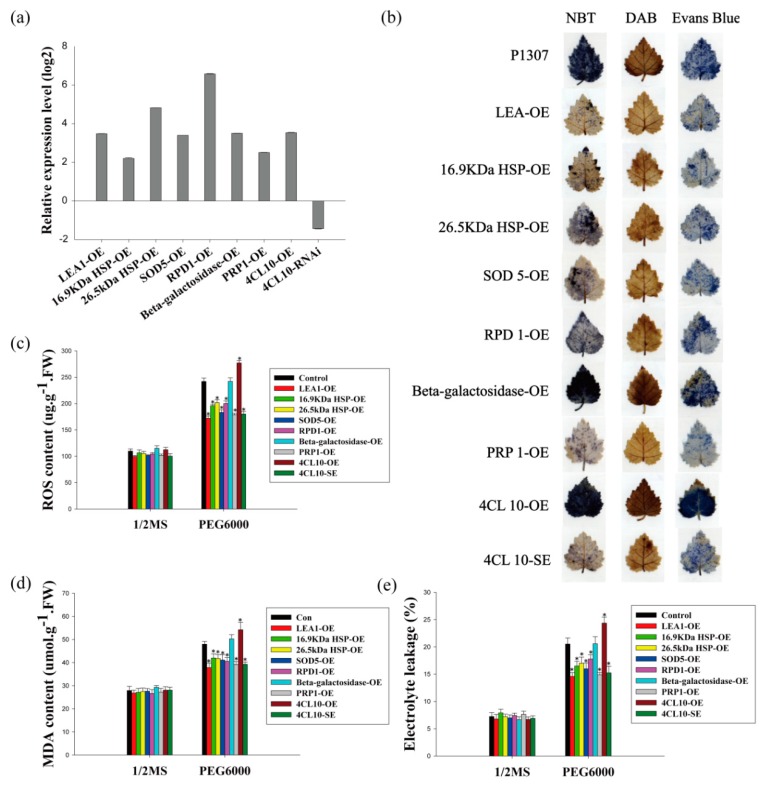
Analysis of the drought stress tolerance of several BpERF2 or BpMYB102 target genes. The plants were transiently transformed with several target genes of BpERF2 and BpMYB102 for overexpression, and empty p1307-myc was transiently transformed into plants as the control. (**a**) Analysis of the expression of the transgenes in transiently transformed plants using qRT-PCR. (**b**) NBT, DAB, and Evans blue staining analysis. (**c**–**e**) Comparison of ROS content (**c**), MDA content (**d**), and electrolyte leakage (**e**) among the studied plants under normal or drought stress conditions. Asterisks indicate a significant difference between treatment and control plants (*p* < 0.05).

Among the target genes directly or indirectly regulated by BpERF2 or BpMYB102 confirmed here, eight genes (*LEA1*, *16.9 kDa HSP*, *26.5 kDa HSP*, *SOD5*, *RPD1*, *Beta-galactosidase*, *PRP1*, and *4CL10*) were chosen to further study their role in the response to drought stress. These genes were transiently overexpressed in birch using a transient transformation method. Since plants overexpressing *BpMYB102* had a down-regulated expression of *4CL10*, we transiently knocked down *4CL10* in birch by transformation with an RNAi construct (*RNAi:4CL10*). The expression levels studied using qRT-PCR of these transgenes increased by 7–96-fold in the transformed birch plants, and the expression of *4CL10* decreased by 63% in the RNAi-transformed birch plants when compared with the control plants (Figure 6a). These results indicated that these genes were successfully overexpressed or silenced in birch, and the plants were suitable for further study.

Among these studied genes, six of them (*LEA1*, *16.9 kDa HSP*, *26.5 kDa HSP*, *SOD5*, *RPD1*, and *PRP*1) appeared to increase plant tolerance to drought stress on the basis of the following observations. (1) The ROS content in plants overexpressing these genes was 71–83% lower than that in the control plants under drought stress conditions (Figure 6c), and this is consistent with DAB and NBT staining (Figure 6b). (2) The MDA content in the plants overexpressing these genes decreased by 11–23% when compared with the control plants (Figure 6d).(3) The electrolyte leakage rates in the plants overexpressing these genes were reduced by 13–29% when compared with the control plants (Figure 6e), and this is in accordance with the Evans blue staining results (Figure 6b). (4) The overexpression line of *4CL10* displayed increased electrolyte leakage rate (Figure 6e), ROS level (Figure 6c), and MDA content (Figure 6d) when compared with the control plants, and these findings were further confirmed by DAB, NBT, and Evans blue staining (Figure 6b), which showed the plants’ decreased tolerance to drought. Conversely, RNAi silencing of *4CL10* displayed decreased electrolyte leakage rate (Figure 6e), ROS level (Figure 6c), and MDA content (Figure 6d) when compared with the control plants, and the RNAi-silenced plants showed improved drought stress tolerance. These results imply that 4CL10 is a negative regulator in drought tolerance, and its expression decreases under drought stress. On the other hand, the overexpression of Beta-galactosidase failed to alter the electrolyte leakage rates, ROS level, and MDA content, suggesting that it does not contribute to tolerance to drought stress.

### 2.5. The Gene Expression Regulatory Network of Birch in Response to Drought

**Figure 7:**
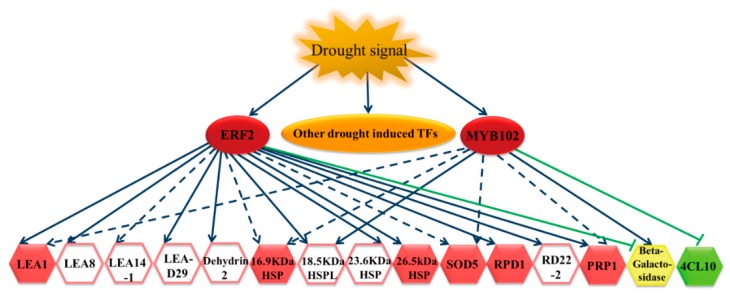
The regulatory network of BpERF2 and BpMYB102 in response to drought stress. Drought stress induces a series of transcription factors, including BpERF2 and BpMYB102, and the induced BpERF2 and BpMYB102 positively or negatively regulate a series of genes to improve drought tolerance. The solid blue lines indicate that BpERF2 or BpMYB102 directly regulates or induces the target gene expression; the solid green lines indicate that BpERF2 or BpMYB102 directly regulates or inhibits the target gene expression; the dotted lines indicate indirect regulation. The red hexagons highlight genes that were confirmed to confer drought tolerance to transgenic plants; the green hexagon indicates a gene that is related to drought sensitivity; the yellow hexagon indicates a gene that is not involved in drought tolerance; the hexagon without color shows genes that were not studied for their function in drought stress here.

Our results showed that BpERF2 and BpMYB102 could be induced by drought stress. After their induction, BpERF2 and BpMYB102 directly and indirectly regulated a series of genes. For instance, BpERF2 directly regulated genes in the LEA family, HSP family, *RPD1*, *RD22-2*, *PRP1*, *Beta-galactosidase*, and *4CL10*, and it indirectly induced genes such as *SOD5* and stress-related genes. At the same time, BpMYB102 directly regulated genes such as *18.5 kDa HSPL*, *Beta-galactosidase*, and *4CL10*, and it indirectly regulated genes such as *LEA1*, *16.9 kDa HSP*, *SOD5*, and *PRP1*. In addition, many of these target genes, such as *LEA1*, *HSPs*, *SOD5*, *RPD1*, *PRP1*, and *4CL10*, are involved in drought stress tolerance. Therefore, these results suggest that BpERF2 and BpMYB102 are important transcription factors in the drought response and can regulate their target genes to improve drought stress tolerance (Figure 7).

## 3. Discussion

In this study, we used the transcriptome of birch to conduct genome-wide screening for genes involved in the response to drought stress tolerance. We also combined the transcriptome with the transient transformation method to identify genes involved in drought stress tolerance and build a regulatory network. Our results show that the ERF and MYB families may play important roles in drought stress tolerance in birch because both the ERF and MYB family transcription factors were abundantly expressed among the drought-induced DGEs (Figure 2a). Under drought stress conditions, we found that some members of the LEA and HSP families were significantly induced in birch (Table S4), suggesting that they play roles in the drought response. The LEA family, including dehydrin, is a hyper-hydrophilic protein family comprising proteins that accumulate under cellular dehydration conditions and play important roles in drought tolerance [17,18,19]. HSPs are a class of molecular chaperones that are ubiquitously expressed and play a key role in the abiotic stress response [20]. Plants expressing HSPs have previously demonstrated increased drought tolerance [21]. The results of further analyses show that many drought-induced LEAs and HSPs were directly or indirectly regulated by BpERF2, but only a few of these drought-induced LEAs and HSPs were regulated by BpMYB102 (Figure 7). Therefore, BpERF2 plays quite an important role in regulating LEA and HSP family genes to improve drought stress tolerance.

In the present study, some genes were functionally characterized to determine their role in drought tolerance; in particular, those genes that have been little studied for their involvement in abiotic stress tolerance were identified here, including pathogenesis-related (PR) protein and the 4-Coumarate-CoA Ligase (4CL) gene, which play roles in drought tolerance. *RPD1* is a plant-specific gene that plays a role in maintaining active cell proliferation, especially when the cycle of cell division is rapid [22]. In addition, RPD1 was also found to be involved in the generation of adventitious roots in response to exogenous auxins [23]. However, there have been few reports about RPD1 and its role in drought stress tolerance. Our results show that a *Root Primordium Defective 1* gene (*BpRPD1*) from birch could be directly regulated by BpERF2 (Figure 4d,e). The results suggest that drought stress conditions induce BpERF2, which then regulates the expression of the *BpRPD1* gene, and the expression of the *BpRPD1* gene improves drought tolerance (Figure 6). Therefore, BpRPD1, mediated by BpERF2, plays a role in drought stress tolerance in birch.

PR proteins are low molecular weight proteins that are induced by various biotic and abiotic stresses in plants and are quite important in plant defense mechanisms [24]. Many PR proteins display enzymatic activity, such as glucanases [25], protease inhibitors [26], peroxidase [27], and chitinases [28], and these activities vastly improve disease resistance in plants. However, few studies have been conducted on their role in drought tolerance. Our results show that the *BpPRP1* gene from birch played a positive role in drought stress tolerance (Figure 6), and it was directly regulated by BpERF2 and indirectly regulated by BpMYB102 when birch was exposed to drought stress conditions (Figure 4; Figure 5). Therefore, under drought stress conditions, both BpERF2 and BpMYB102 are induced, and these two TFs together positively regulate the expression of *BpPRP1*, and the increased expression of BpPRP1 contributes to improved drought tolerance.

The 4CL protein contributes to the channelization of the flux of different phenylpropanoid biosynthetic pathways by catalyzing the formation of the CoA ester, and it also plays a role in the biosynthesis of flavonoid and lignin [29]. These 4CL products control various physiological functions and also play roles in the improvement of plant adaptations to environmental stress [30]. Our results show that the birch *Bp4CL10* gene played a negative role in drought tolerance (Figure 6), and BpMYB102 directly regulated the expression of the *Bp4CL10* gene (Figure 5a,e). These results together suggest that drought stress induces the expression of BpMYB102, which then down-regulates the expression of *Bp4CL10*, and the inhibition of Bp4CL10 increases drought tolerance.

In conclusion, on the basis of the RNA-Seq results, we selected two TFs that may play important roles in the drought tolerance of birch for further study. We characterized the function of these two TFs in drought tolerance, identified their target genes, and further characterized the stress tolerance of several target genes. RNA-Seq, combined with the molecular and physiological assay based on a transient genetic transformation platform, is effective for building a regulatory network and characterizing stress tolerance genes. This transient transformation method can be used in different plant species, including those that do not have a stable transformation system. Therefore, the strategy used in this study for the investigation of a regulatory network in response to abiotic stress has a wide range of applications.

## 4. Materials and Methods

### 4.1. Plant Materials and Growth Conditions

*B. platyphylla* seedlings used for the RNA-Seq assay were grown in soil in a greenhouse under controlled conditions, including a 14 h light/10 h dark photocycle, a stable temperature of 24 °C, 70–75% relative humidity, and sufficient watering. To induce drought stress, 3-month-old seedlings had their water supply stopped for 120 h. Meanwhile, seedlings that continued to be well-watered with freshwater were used as controls. The birch plants (sample size of 20 seedlings) were harvested and pooled for RNA isolation, and three independent biological replications were performed.

To culture the birch seedlings in a tube, birch seeds were surface-sterilized in 10% (*v*/*v*) sodium hypochlorite for 10 min followed by washing with sterile water five times. They were then grown in tissue culture bottles containing Woody Plant Medium (WPM) (with 2.5% (*w*/*v*) sucrose and 0.6% (*w*/*v*) agar) in a culture room at a constant temperature of 24 °C with a 14/10 h light/dark photoperiod and 70–75% relative humidity.

### 4.2. Transcriptome Profile of Birch in Response to Drought Stress

Total RNA was isolated independently from six samples (three drought and three control treatments) using the cetrimonium bromide (CTAB) method. Then, mRNA was purified from the total RNA using poly-T oligo-attached magnetic beads. Sequencing libraries were generated using the NEBNext^®^Ultra™ RNA Library Prep Kit for Illumina^®^ (NEB, Ipswich, MA, USA) following the manufacturer’s recommendations, and index codes were added to attribute sequences to each sample. After cluster generation, the library preparations were sequenced on an Illumina HiSeq platform, and paired-end reads were generated. Raw data (raw reads) were first processed through in-house Perl scripts to obtain clean data (clean reads). At the same time, the Q20, Q30, GC content, and sequence duplication level of the clean data were calculated. Gene expression levels were estimated by fragments per kilobase of transcript per million fragments mapped. Differential expression analysis of two conditions was performed using the DESeq R package (1.10.1). Genes with an adjusted *p*-value < 0.05 found by DESeq were identified as differentially expressed. GO enrichment analysis of the DEGs was implemented by the GOseq R packages, and Wallenius non-central hypergeometric distribution [31] was used to adjust for gene length bias in DEGs.

### 4.3. Construction of Plant Expression Vectors and Transient Transformation

The intact coding sequence (CDS) of the studied transcription factors were fused in frame with the C-terminus of 3 × Flag tag under the control of the CaMV 35S promoter in the p1307-Flag plant expression vector. The intact CDS of the studied functional genes replaced 5 × Myc tags and cloned into the p1307-myc vector under the control of the CaMV 35S promoter to generate the plant expression vectors. To construct the RNAi silence vector, an inverted repeat fragment cDNA of a gene 249 bp in length was cloned into the RNAi vector pFGC5941 on the two sides of the *Chalcone Synthase A* (*CHSA*) intron. All constructs were confirmed by DNA sequencing and transferred into *Agrobacterium* EHA105; all primers used for construction are shown in Supplementary Table S1. Transient transformation was performed as described by Zang et al. [32]. The colonies of *Agrobacterium tumefaciens* EHA105 harboring the studied constructs were cultured, harvested by centrifugation, and adjusted to an OD_600_ of 0.8 with 50 mL of transformation solution [1/2 MS  +  2.5% (*w*/*v*) sucrose  +  100 μM acetosyringone  +  Tween 20 (0.01%, *v*/*v*), pH 5.8]. Whole plants under tissue culture conditions were soaked in the transformation solution with shaking at 100 rpm at room temperature. After 2 h of culture, the plants were taken out of the transformation solution, quickly washed with distilled water twice, and wiped with sterile filter paper to remove the excess water. The plants were vertically planted on 1/2 MS solid medium [1/2 MS + 1% (*w*/*v*) sucrose + 120 μM acetosyringone, pH 5.8] for recovery, and genetic transformation occurred during this period. After culturing for 48 h, the transgene began to be highly expressed in plants and could be used for subsequent studies. The plants transformed with empty p1307-Flag or p1307-myc were used as the controls (Con).

### 4.4. Identification of Drought Stress Tolerance and Physiological Changes

After transient transformation for 36 h, the transformed plants were divided into two groups: one group was transferred to the WPM solid medium (with 20% PEG6000 overlay solution added) for 48 h for drought treatment, and the other group was grown in normal WPM medium as the control.

Whole plants were used to determine leaf water content, electrolyte leakage, total chlorophyll content, MDA content, and ROS content. Leaves were detached from plants for staining with nitroblue tetrazolium (NBT), 3,3-diaminobenzidine (DAB), and Evans blue. For soil water content and leaf water content measurements, soil and detached leaves were weighed immediately (fresh weight, FW) and then oven-dried at 80 °C to a constant dry weight (DW). Water content (WC) was measured according to the formula WC (%) = (FW − DW)/DW × 100%. Electrolyte leakage was determined following the method described by Wang et al. [33]. The chlorophyll content was measured according to Gitelson et al. [34]. MDA content measurements were performed following Wang et al. [35]. ROS production was assessed using a commercially available kit from Nanjing Senbeijia Bioengineering Institute (Nanjing, China). O^2−^ accumulation, H_2_O_2_ accumulation, and cell death in the leaves were assayed by NBT, DAB, and Evans blue staining, respectively. NBT and DAB in situ staining were performed according to Zhang et al. [36], and Evans blue in situ staining followed the procedures described by Kim et al. [37]. At least 10 plantlets were included in each sample, and three independent biological replicates were performed to ensure the accuracy of the analyses.

### 4.5. Real-Time RT-PCR Analysis

Total RNA was isolated from *B. platyphyll*a using the CTAB method [38] and digested with DNaseI to remove DNA contamination. One microgram of total RNA from each sample was reverse transcribed into cDNA using oligo(dT) primers with the PrimescriptTMRT reagent kit (Takara). The resulting cDNA product was diluted 10-fold with ultrapure water (Milli-Q) as the PCR template. α-Tubulin (GenBank number: FG067376) was used as the internal reference to normalize the number of templates used in the PCR reaction. Real-time PCR was performed using an MJ Research OpticonTM^2^ instrument (Bio-Rad, Hercules, CA, USA) under the following conditions: 94 °C for 60 s; 45 cycles at 94 °C for 10 s, 59 °C for 20 s, 72 °C for 30 s; and 80 °C for 1 s for plate reading. The reaction mixture contained 10 μL of SYBR Green Real-time PCR Master Mix (Toyobo, Osaka, Japan), 0.5 μM of each forward and reverse primer, and 2 μL of cDNA template in a volume of 20 μL. A melting curve was generated to assess the purity of the amplified products. All experiments were carried out with three biological replicates, and the relative expression levels were determined using the 2^−ΔΔ*C*t^ method [39]. All primers used in this study are shown in Supplementary Table S2.

### 4.6. Chromatin Immunoprecipitation (ChIP) Analysis

The transiently transformed plants, including the plants transformed with *35S:Flag-BpERF2* and *35S:Flag-BpMYB102*, were used for the ChIP assay. The ChIP experiment was performed according to Haring et al. [40]. Briefly, protein and DNA were cross-linked with 1% formaldehyde. The cross-linked chromatins were fragmented into 0.5–0.8 kb by sonication, and 1/10 volume was saved as the input control. The remaining material was separated into two equal aliquots: one part was incubated with the anti-Flag antibody for immunoprecipitation; the other part, which was incubated without the anti-Flag antibody, was used as the negative control. The antibody-bound complex was precipitated by protein A agarose beads, and the DNA fragments were released by incubating the cross-linked complex at 65 °C for 5 h. Enriched DNA fragments were purified by chloroform extraction. Real-time PCR was performed with the same cycling parameters and reaction mixture as described for Real-time RT-PCR analysis. The primers used for ChIP-PCR are shown in Supplementary Table S3.

### 4.7. Statistical Analyses

Statistical analyses were carried out using SPSS 16.0 (SPSS Inc., Chicago, III, USA) software. Data were compared using Student’s *t*-test. Differences were considered to be significant if *p* < 0.05.

### 4.8. Data Availability

The nucleotide sequence data in this study were submitted to GenBank (https://www.ncbi.nlm.nih.gov/WebSub/?tool=genbank). The GenBank accession numbers are as follows: BpERF2 (MK112037), BpMYB102 (MK112038), BpLEA1 (MK112039), Bp16.9kDa HSP (MK112040), Bp26.5kDa HSP (MK112041), BpSOD5 (MK112042), BpRPD1 (MK112043), BpBeta-galactosidase (MK112044), BpPRP1 (MK112045), Bp4CL10 (MK112046).

## Supplementary Materials

Supplementary materials can be found at https://www.mdpi.com/1422-0067/20/12/3071/s1. Table S1: List of primers used for construction. Table S2: List of primers used for qRT-PCR experiments. Table S3: List of primers used for ChIP. Table S4: Differential expression gene data. Figure S1: Transcriptome data analysis. (**a**) The correlation coefficients between the different samples; (**b**) volcano plot of the differentially expressed genes; (**c**) functional analysis of the differentially expressed genes using GO term annotations.

## Abbreviations

TFs	Transcription factors
DEGs	Differentially expressed genes
LEA	Late embryogenesis abundant
HSP	Heat shock protein
PRP1	Pathogenesis-related protein 1
RPD1	Root primordium defective 1
PRP1	Pathogenesis-related protein 1
4CL10	4-Coumarate:Coenzyme A Ligase 10
WPM	Woody Plant Medium
NBT	Nitroblue tetrazolium
DAB	3,3-diaminobenzidine
MDA	Malondialdehyde
